# Shape transition from elliptical to cylindrical membrane tubes induced by chiral crescent-shaped protein rods

**DOI:** 10.1038/s41598-019-48102-7

**Published:** 2019-08-13

**Authors:** Hiroshi Noguchi

**Affiliations:** Institute for Solid State Physics, University of Tokyo, Kashiwa, Chiba 277-8581 Japan

**Keywords:** Computational biophysics, Membrane structure and assembly, Biological physics

## Abstract

Proteins often form chiral assembly structures on a biomembrane. However, the role of the chirality in the interaction with an achiral membrane is poorly understood. Here, we report how chirality of crescent-shaped protein rods changes their assembly and tubulation using meshless membrane simulations. The achiral rods deformed the membrane tube into an elliptical shape by stabilizing the edges of the ellipse. In contrast, the chiral rods formed a helical assembly that generated a cylindrical membrane tube with a constant radius in addition to the elliptical tube. This helical assembly could be further stabilized by the direct side-to-side attraction between the protein rods. The chirality also promotes the tubulation from a flat membrane. These results agree with experimental findings of the constant radius of membrane tubules induced by the Bin/Amphiphysin/Rvs (BAR) superfamily proteins.

## Introduction

The molecular chirality and single-handedness have been under strong selection in evolution^1^. Proteins consist of L-amino acids and form chiral structures from a local secondary structure, the right-handed *α*-helix, to micrometer-scale assemblies such as a microtubule. This chirality is important for the recognition of molecular binding, and the interactions between chiral molecules are often governed by a geometrical match. The molecular chirality is crucial for the binding affinity to DNA double helices^2^. For example, a right-handed molecule strongly binds to the minor groove of the DNA helix, whereas binding of the left-handed form may be prevented due to excluded volume interactions. Moreover, helical self-assembled fibers are formed by various types of chiral biomolecules and synthetic molecules^3–6^. However, the role of chirality in the interaction between chiral and achiral structures remains poorly understood in comparison with the interactions between chiral structures. Accordingly, the aim of this study is to clarify the mechanisms underlying the chirality of membrane-binding proteins in the interaction with deformable achiral membranes.

In living cells, lipid membranes are maintained in a fluid phase, in which the chiral molecular interactions between chiral lipids are smeared out so that the chirality does not appear in membrane dynamics. However, in a gel phase, chiral amphiphiles can form helical-ribbon structures, which transform to fluid vesicles at high temperature^7–9^. Here, we consider a biomembrane as a two-dimensionally isotropic achiral fluid membrane.

Many proteins are known to bind biomembranes and consequently reshape them^10–15^. Such proteins are often found in a helical assembly formation. For example, dynamins assemble on the neck of a clathrin-coated membrane bud and form a helix surrounding the neck to induce the membrane fission^16^. Proteins containing a Bin/Amphiphysin/Rvs (BAR) domain, which consists of a banana-shaped dimer, bind the membrane and bend it along the BAR domain axis via scaffolding^17–21^. The BAR domains have chirality, and helical alignment on the membrane tubes has been observed by electron microscopy^20,22,23^. However, the role of this helicity is not known. At low concentration, BAR-containing proteins solely bind the membrane with maintained ability as membrane scaffolding^24^. The two-dimensional crystal alignment of the BAR domains induces substantially greater bending ability; however, it is unclear whether a helical structure is essential for this effect, and if achiral crystal assembly might yield the same degree of bending. Since it is nearly impossible to separate the chirality and regularity of a protein assembly experimentally, here, the general effects of the chirality were investigated using numerical simulations in which chirality can be readily switched on and off. The protein-rod assembly on a membrane tube in thermal equilibrium and tubulation dynamics from a flat membrane were examined.

The attractive interactions between specific sites of the BAR domains were then considered in terms of elucidating the origin of the regular assembly^20,22,23,25^. The I-BAR protein, Ivy1p, forms a filament by side-to-side attractions, even in the absence of membranes^25^. N-terminal H0 helices of endophilin have a hook-like shape and form dimers in the helical assembly^20^. Therefore, the effects of the side-to-side attraction of chiral proteins on membrane tubulation were also investigated.

The binding of BAR proteins to the membranes and resulting shape deformation have previously been simulated using various approaches from atomistic molecular dynamics to mesoscale coarse-grained models^26–37^. Although the atomistic and coarse-grained molecular models of the BAR domains^26–30^ have chirality, the chirality effects have not been investigated to date.

In this study, we examined the effects of such chirality in crescent-shaped protein rods on the shape deformation of membrane tubes and tubulation from a flat membrane using coarse-grained membrane simulations. Several types of simulation models have been developed to investigate membranes^38–40^. Since we investigated large-scale membrane dynamics, we employed a meshless membrane model, in which membrane particles self-assemble into a membrane and the membrane elastic properties are highly tunable^40–42^. A tubular membrane is a well-developed experimental setup^12^; the tubular tether membrane is extended from a vesicle by optical tweezers so that the tube radius is controllable by manipulating the mechanical force. We examined how the rod chirality changes the assembly structure and shapes of the membrane tubes. The chiral rods behaved similarly to the achiral rods at a low curvature *C*_rod_ but formed a helical cylinder at a high curvature. This circular tube could not be induced by the achiral rods. In addition, the side-to-side attraction between the chiral rods reinforces this cylinder formation. The chiral rods generate tubulation faster than the achiral rods.

## Results

### Rod assembly on membrane tube

**Figure 1:**
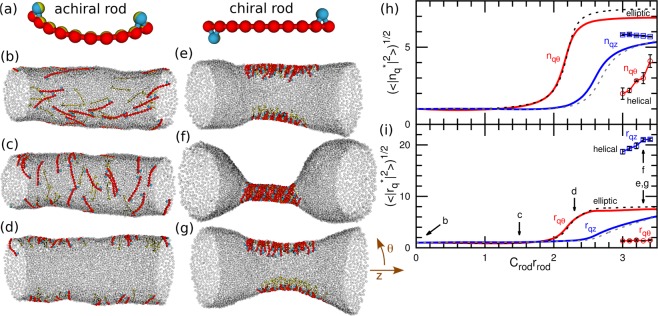
Membrane tube deformation induced by chiral and achiral protein rods without direct attraction between the rods at *R*_cyl_/*r*_rod_ = 1.31 and *N* = 4800. (**a**) Protein models for achiral and chiral crescent-shaped rods. (**b**–**g**) Snapshots at (**b**) *C*_rod_*r*_rod_ = 0, (**c**) 1.5, and (**d**) 2.3, and **(e**,**f**) 3.3 for the chiral rods; and (**g**) *C*_rod_*r*_rod_ = 3.3 for the achiral rods. The membrane particles are displayed as transparent gray spheres. (**h**,**i**) Fourier amplitudes of (**h**) rod densities and (**i**) membrane shapes. The solid and dashed lines represent the data for REMD of the chiral and achiral protein rods, respectively. The circles and squares with solid lines represent the *qθ* and *qz* modes, respectively, for the canonical simulations of the helical rod-assembly as shown in (**f**). The Fourier amplitudes are normalized by the values at *C*_rod_ = 0 (denoted by the superscript *). The error bars are displayed only for the canonical simulations. The errors in REMD are smaller than the thickness of the lines.

The fluid membrane is modeled as single-layer self-assembled particles. A BAR protein is assumed to be strongly adsorbed onto the membrane and not to be detached from the membrane; thus, the BAR and the bound membrane are modeled together as a chiral or achiral crescent-shaped rod of the length of *r*_rod_ with the spontaneous curvature of *C*_rod_ along the rod axis. To construct a minimum model of chiral protein rods, our previously reported linear protein model^32–37^ is modified by adding two particles in the right-handed positions as shown in the right panel of Fig. 1(a). As a reference, an achiral rod which two hooks are on the same side is constructed as shown in the left panel of Fig. 1(a).

Short and long membrane tubes with the total number of particles *N* = 4800 and 9600 are simulated with a constant low protein density *ϕ*_rod_ = 0.0833 in which 40 and 80 protein rods are embedded, respectively. Two average radii of the membrane tube *R*_cyl_/*r*_rod_ = 1.18 and 1.31 corresponding to tube length *L*_*z*_/*r*_rod_ = 0.00167*N* and 0.0015*N* are employed, respectively, for both *N* = 4800 and 9600.

Figure 1 shows the rod assembly via membrane-mediated interactions for the achiral and chiral rods. For low rod curvatures of *C*_rod_, there is no quantitative difference observed between chiral and achiral rods. As the rod curvature *C*_rod_ increases, the orientation of the rods rotates from the longitudinal (*z*) direction to the azimuthal (*θ*) direction (Fig. 1(b,c)). With a further increase in *C*_rod_, the membrane deforms into an elliptical tube and the rods accumulate at the edges of the ellipse (Fig. 1(d)). An even further increase in *C*_rod_ induces the rod assembly also in the *z* direction, and the rest of the tube forms a more rounded shape (Fig. 1(e,g)). The amplitudes of the Fourier modes of the rod density and membrane shape are shown in Fig. 1(h,i), respectively: ${r}_{qz}=\mathrm{(1}/N){\sum }_{i}\,{r}_{{\rm{2}}D,i}\exp (\,-\,2\pi {z}_{i}i/{L}_{z})$ and ${r}_{q\theta }=\mathrm{(1}/N){\sum }_{i}\,{r}_{{\rm{2}}D,i}\exp (\,-\,2{\theta }_{i}{\rm{i}})$ where ${r}_{2{\rm{D}},i}^{2}={x}_{i}^{2}+{y}_{i}^{2}$ and ${\theta }_{i}={\tan }^{-1}({x}_{i}/{y}_{i})$. The subscripts *qz* and *qθ* represent the lowest Fourier modes along the *z* and *θ* directions, respectively. Both amplitudes of the membrane shape *r*_*qθ*_ and rod density *n*_*qθ*_ along the *θ* direction increase together, and the amplitudes of *r*_*qz*_ and rod density *n*_*qθ*_ along the *z* direction increase later. Thus, the membrane deformation and rod assembly independently occur in the longitudinal and azimuthal directions. The details of these shape changes of the achiral rods are described in our previous papers^32,33,35^. The excluded volume interactions of the hooks slightly increase the assembly curvatures *C*_rod_ as shown in the Supplemental Material.

By contrast, at high curvatures of *C*_rod_, it is found that the chiral rods assemble into helical strips and deform the membrane into a cylindrical shape, as shown in Fig. 1(f). This helical assembly coexists with the elliptical assembly for the short and wide membrane tube (*N* = 4800 and *R*_cyl_/*r*_rod_ = 1.31), but the elliptical assembly becomes unstable and spontaneously transforms into the helical assembly for the longer or narrower tubes (*N* = 9600 or *N* = 4800 and *R*_cyl_/*r*_rod_ = 1.18), as shown in Supplemental Movie 1. This shape transition causes the rods to be packed in the helical assembly so that the membrane becomes axisymmetric and narrow (Fig. 1(h,i)). In the elliptical tubes, the chiral rods form oligomers with a helical-strip shape (Fig. 1(e)) but the oligomer size is restricted by the elliptic edge, since the large oligomers stick out from the highly curved region of the edge. By removing the flat region of the elliptical tube, the rods can form a single large assembly on the circular tube. However, the achiral rods do not form this helical assembly. Thus, the chirality appears to be essential to form this circular tube formation.

**Figure 2:**
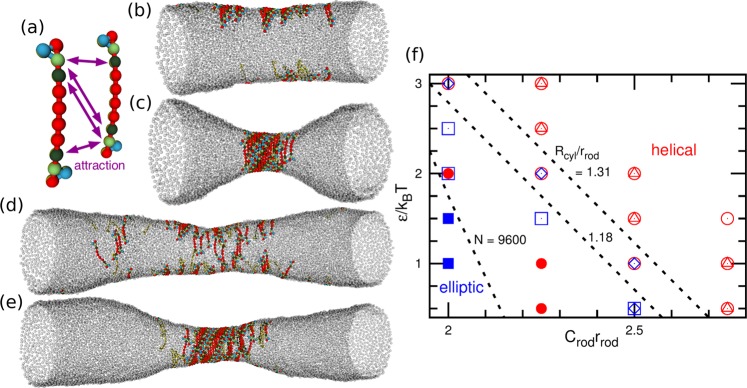
Membrane tube deformation induced by chiral protein rods with direct attraction between rods. (**a**) Protein model. (**b**–**e**) Snapshots of membrane tubes at *R*_cyl_/*r*_rod_ = 1.31. (**b**) *ε*/*k*_B_*T* = 2 and (**c**) 2.5 at *C*_rod_*r*_rod_ = 2.25 and *N* = 4800. (**d**) *ε*/*k*_B_*T* = 1.5 and (**e**) 2 at *C*_rod_*r*_rod_ = 2 and *N* = 9600. (**f**) Phase diagram for rod assembly of the elliptical tube and helical-cylinder shape. The open circles (triangles) and squares (diamonds) represent the elliptical and helical tubes at *R*_cyl_/*r*_rod_ = 1.18 (1.31) for *N* = 4800, respectively. The closed circles and squares are for *N* = 9600 at both *R*_cyl_/*r*_rod_ = 1.18 and 1.31. The dashed lines indicate the phase boundary.

Although it has been generally accepted that the direct attractive interactions between specific sites of the BAR domains are essential for the helical assembly^20^, the results of the present simulation revealed that these interactions are in fact not necessary. Instead, the direct attractions between the protein segments largely promote the assembly. To further clarify these attraction effects, we added the direct attractive interaction between the second and third particles from both rod ends as shown in Fig. 2. The rods gain this side-to-side attraction when they assemble into a helical strip. Thus, the helical-cylinder-shaped rod-assembly can be stabilized by this attraction (Fig. 2(c,e) and Supplemental Movie 2). On the other hand, in the elliptical membrane tube, the rods can form only oligomers as observed for the rods without the direct attractions (Fig. 2(b)). Therefore, this direct side-to-side attraction enhances the formation of the helical assembly, as shown in the phase diagram of Fig. 2(f). The greater attraction then induces the helical formation at lower curvatures of *C*_rod_. This transition point slightly depends on the average tube radius *R*_cyl_ for the short tube (*N* = 4800), while no such radius dependence is obtained for the long tube (*N* = 9600). Thus, the radius *R*_cyl_ has only slight effects on the rod assembly. We also confirmed that other combinations of the attractive segment pairs and hook position can similarly stabilize helical assemblies as shown in the Supplemental Material.

**Figure 3:**
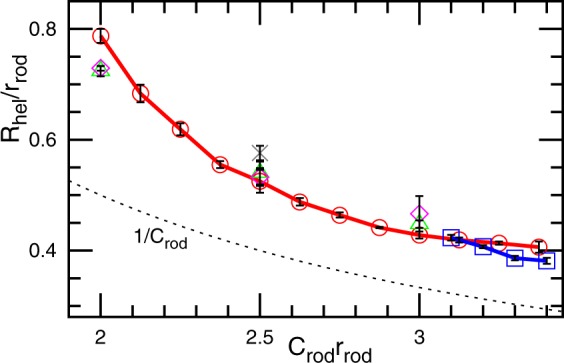
Radius *R*_hel_ of the rod assembly of the helical-cylinder shape for the chiral rods with (⚬, Δ, ◊) and without (◽, ×) direct side-to-side attractive interactions. The circles and squares represent *R*_hel_ of the rods at *R*_cyl_/*r*_rod_ = 1.18 and *N* = 4800. The triangles and diamonds are for *R*_cyl_/*r*_rod_ = 1.18 and 1.31, respectively, at *N* = 9600. For the side-to-side attraction, *ε*/*k*_B_*T* = 3 is used. The cross represents *R*_hel_ of the tubules protruded from flat membranes at *ϕ*_rod_ = 0.4. The dashed line shows the curvature radius of the rods 1/*C*_rod_.

The radius *R*_hel_ of the helical assembly is a monotonically decreasing function of *C*_rod_ and exhibits little dependence on the other parameters as shown in Fig. 3. The radius *R*_hel_ is calculated by the least-squares fit for the slice of the middle region with a width of 0.4*r*_rod_ as ${R}_{{\rm{hel}}}=(1/{N}_{{\rm{sl}}}){\sum }_{i}\,|{{\rm{r}}}_{2{\rm{D}},i}-{{\rm{r}}}_{2{\rm{D}},{\rm{g}}}|$, where *N*_sl_ is the number of the fit particles and r_2D,g_ is the center of the mass projected on the *xy* plane. Since the rods are not completely rigid, *R*_hel_ is slightly greater than the preferred radius of the rod curvature 1/*C*_rod_. For the elliptical tube, the mean tube radius can be largely varied, since the flat region of the ellipse can be increased by the addition of protein-unbound membranes. In contrast, the tube radius of the helical rod-assembly is uniquely determined by the protein. This finding shows good agreement with experimental evidence that each type of BAR protein typically generates a constant radius of the membrane tubules^17–21^.

### Tubulation from flat membrane

**Figure 4:**
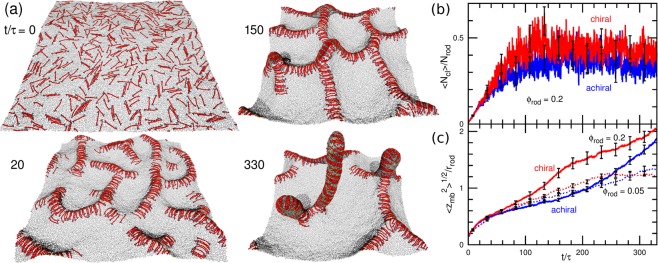
Tubulation from tensionless flat tensionless membrane. (**a**) Sequential snapshots for the high rod density *ϕ*_rod_ = 0.2 and *C*_rod_*r*_rod_ = 2.5 at *t*/*τ* = 0, 20, 150, and 330. (**b**,**c**) Time development of (**b**) mean cluster size $\langle {N}_{{\rm{cl}}}\rangle$ and (**c**) vertical membrane span ${\langle {z}_{{\rm{mb}}}^{2}\rangle }^{\mathrm{1/2}}$. The red and blue lines show the data for the chiral and achiral rods, respectively. The solid lines show the data for *ϕ*_rod_ = 0.2 and *C*_rod_*r*_rod_ = 2.5. The dashed lines in (**c**) show the data for the low density *ϕ*_rod_ = 0.05 and *C*_rod_*r*_rod_ = 3.

Next, we describe the chirality effects on the tubulation dynamics. The chirality does not induce qualitative changes in the tubulation processes reported in ref.^34,37^ but accelerates the tubule formation. For a high rod density *ϕ*_rod_ = 0.4, the rods first form a percolated two-dimensional (2D) network on the membrane and then tubules protrude from the branching points of the network as shown in Fig. 4(a) and Supplemental Movie 3. The assembly speed into the network is almost identical between chiral and achiral rods and the time developments of mean cluster size in Fig. 4(b) are overlapped. However, the tubules are formed faster by the chiral rods than by the achiral rods (see Fig. 4(c)). The chiral rod assembly tends to helically wrap the membrane bump at the branching points and it helps the formation of the tubule nucleus. The tubules have a cylindrical shape connected with a disk-shaped tip (see Fig. S4 in the Supplemental Material). The tube radius coincides well with that obtained in the ellipse-to-cylinder transition in the membrane tubes (see Fig. 3). The disk shape of the tip is reasonable since it can have lower bending energy than a hemispherical tip.

For a low rod density *ϕ*_rod_ = 0.05, the tubulation occurs without a formation of the 2D network (see Supplemental Movie 4). The chirality weakly promotes the tubulation (see the dashed lines in Fig. 4(c)). Since the chiral rods can assemble more compactly than the achiral rods, the chiral-rod assembly can bend the membrane slightly more strongly so that the rod assemblies protrude slightly faster and slightly more tubules are eventually formed. At both low and high rod densities, the helical rod assembly is formed in growing tubules. Thus, the shape transition from the two-lane elliptical tube to helical circular tube also occurs during the dynamic tubulation process.

## Discussion

The present coarse-grained simulation demonstrates that the chirality of proteins induces helical rod assembly on a cylindrical membrane of a constant radius and induces faster tubulation. The side-to-side direct attraction between proteins stabilizes this assembly. Although the proteins can still induce membrane tubules without the chirality, the shape is elliptical and the radius is not constant. The helical interaction induces the protein assembly not only in the side-to-side direction but also in the tip-to-tip direction leading to the formation of a circular tube. The helical assembly also accelerates the tubulation dynamics, in particular, tubule protrusion from the branching points of the 2D network protein assembly. These scenarios seem to be more efficient than the case, in which different assembly mechanisms are employed for the side and tip directions. This type of helical interaction may also play an important role in other protein assemblies, such as those occurring on biomembranes including dynamins^16^ and ESCRT^43^.

Here, we consider a simple achiral side-to-side attractive interaction between protein rods and the chirality is only generated by the excluded volume of the right-handed hook particles in order to clarify the general aspects of the chirality effects. Although the attraction strength to induce the helical assembly is varied by the change in the positions of the hook and attraction segments, the essential dynamics itself does not change. Hence, it is robust to such structure modifications. However, BAR proteins often have multiple interaction sites, and some of these proteins also exhibit tip-to-tip interactions^22,44^, which likely connects two helical strips to stabilize the assembly. The F-BAR protein, Pacsin, induces membrane tubes over a wide diameter range and is considered to have two types of assembly structures^45^. Determining the effects of such specific interactions on the membrane tubulation would therefore be an interesting problem for further investigations.

## Methods

Since the details of the meshless membrane model and achiral protein rods are described in ref.^32^ and refs^32,35^, respectively, we briefly describe the model here.

The position and orientation vectors of the *i*-th particle are ***r***_*i*_ and ***u***_*i*_, respectively. The membrane particles interact with each other via a potential $U={U}_{{\rm{rep}}}+{{\rm{U}}}_{{\rm{att}}}+{U}_{{\rm{bend}}}+{U}_{{\rm{tilt}}}$, in which *U*_rep_ represents an excluded volume interaction with diameter *σ*, *U*_att_ is the attractive potential to implicitly account for the effects of the solvent, and *U*_bend_ and *U*_tilt_ are the bending and tilt potentials given by ${U}_{{\rm{bend}}}/{k}_{{\rm{B}}}T=({k}_{{\rm{bend}}}/\mathrm{2)}{\sum }_{i < j}\,{({{\boldsymbol{u}}}_{i}-{{\boldsymbol{u}}}_{j}-{C}_{{\rm{bd}}}{\hat{{\boldsymbol{r}}}}_{i,j})}^{2}{w}_{{\rm{cv}}}({r}_{i,j})$ and ${U}_{{\rm{tilt}}}/{k}_{{\rm{B}}}T=({k}_{{\rm{tilt}}}/\mathrm{2)}{\sum }_{i < j}\,[({{\boldsymbol{u}}}_{i}\cdot {\hat{{\boldsymbol{r}}}}_{i,j}{)}^{2}+{({{\boldsymbol{u}}}_{j}\cdot {\hat{{\boldsymbol{r}}}}_{i,j})}^{2}]{w}_{{\rm{cv}}}({r}_{i,j})$, respectively, where ${{\boldsymbol{r}}}_{i,j}={{\boldsymbol{r}}}_{i}-{{\boldsymbol{r}}}_{j}$, ${r}_{i,j}=|{{\boldsymbol{r}}}_{i,j}|$, ${\hat{{\boldsymbol{r}}}}_{i,j}={{\boldsymbol{r}}}_{i,j}/{r}_{i,j}$, *w*_cv_(*r*_*i*,*j*_) is a weight function, and *k*_B_*T* denotes the thermal energy. The spontaneous curvature *C*_0_ of the membrane is given by ${C}_{0}\sigma ={C}_{{\rm{bd}}}/2$^42^. For this study, the parameters *C*_0_ = 0 and *k*_bend_ = *k*_tilt_ = 10 were adopted in all cases except for the membrane particle pairs belonging to the protein rods. The membrane was given mechanical properties that are typical of lipid membranes: the bending rigidity $\kappa /{k}_{{\rm{B}}}T=15\pm 1$, area of the tensionless membrane per particle ${a}_{0}/{\sigma }^{2}=1.2778\pm 0.0002$, and area compression modulus ${K}_{A}{\sigma }^{2}/{k}_{{\rm{B}}}T=83.1\pm 0.4$. The edge line tension ${\rm{\Gamma }}\sigma /{k}_{{\rm{B}}}T=5.73\pm 0.04$ is set to be sufficiently large to prevent membrane rupture in the present simulations.

For modeling the protein rods, 10 membrane particles are linearly connected by the harmonic bond and bending potentials (the rod length *r*_rod_ = 10*σ*)^32^. A relatively stronger bending rigidity, *k*_bend_ = *k*_tilt_ = 80 is used for the protein rods than the membrane, since the protein binding stiffens the membrane. Then, to add chirality, the particles between the first and second particles of both rod ends are right-handedly added in a hook formation and the excluded volumes of these two particles generate the chiral interactions between the rods. For three harmonic bonds to form the triangle including the hook particle, three times greater bond coefficient is used to prevent a flip of the hook particle to the opposite (*i.e*., left-handed) site (see Fig. 1(a)). Some BAR proteins, such as endophilin and APPL1, have a similar hook-like structure^20^. When the direct attraction between segments is added, the excluded potential *U*_rep_ is replaced by the Lennard-Jones (LJ) potential (${U}_{{\rm{LJ}}}=\sum 4\varepsilon [(\sigma /{r}_{ij}{)}^{12}-{(\sigma /{r}_{ij})}^{6})]$) between the second and third particles from both rod ends (Fig. 2(a)).

The curvature of the protein rods is varied from *C*_rod_*r*_rod_ = 0 to 3.5, and zero spontaneous side curvatures^34^ in perpendicular to the rod axis is adopted. These rod length and curvatures are within the typical range of known BAR proteins. The BAR domain length ranges from 13 to 27 nm^18^ and the rod curvatures are varied from negative to positive values. Among the BAR proteins, APPL1 has the maximum curvature reported with a radius of the curvature is 5.5 nm and the length is 17 nm, *i.e*., *C*_rod_*r*_rod_ ≃ 3^46^.

For tubulation simulations, the protein rods are initially equilibrated with the rod curvature *C*_rod_ = 0 in the tensionless flat membrane at *N* = 25600 and then the curvature *C*_rod_ are altered to the target value at *t* = 0.

Molecular dynamics with a Langevin thermostat is employed^42,47^. We use $\tau ={r}_{{\rm{rod}}}^{2}/D$ for the time unit, where *D* is the diffusion coefficient of the membrane particles in the tensionless membrane^35^. In addition to canonical ensemble simulations, replica exchange molecular dynamics (REMD)^48,49^ for the rod curvature *C*_rod_^32^ is used to accurately obtain the thermal equilibrium states for the membrane tubes at *N* = 4800. The error bars are estimated from three or more independent runs for membrane tube simulations and from ten independent runs for flat membrane simulations, respectively.

## Supplementary information

Supplemental Movie 1

Supplemental Movie 2

Supplemental Movie 3

Supplemental Movie 4

Supplementary information
