# Supplementary material for: Shape transition from elliptical to cylindrical membrane tubes induced by chiral crescent-shaped protein rods
Source: Sci Rep. 2019 Aug 13;9:11721. doi: 10.1038/s41598-019-48102-7 (PMC6692377; doi:10.1038/s41598-019-48102-7)
Supplement: Supplementary file 5 — Supplementary information [file 41598_2019_48102_MOESM5_ESM.pdf]

Supplemental Material: Shape transition from elliptical to cylindrical membrane tubes induced by chiral crescent-shaped protein rods

Hiroshi Noguchi
Institute for Solid State Physics, University of Tokyo, Kashiwa, Chiba 277-8581, Japan. e-mail: noguchi@issp.u-tokyo.ac.jp

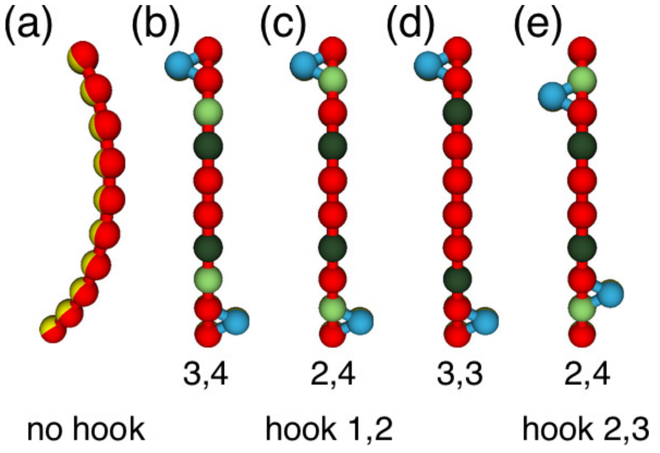

FIG. S1. Protein models. (a) Achiral crescent-shaped rod without hook particles. (b)–(e) Chiral crescent-shaped rod with attractive interaction. The hook particles are connected with (b)–(d) the first and second particles [(e) the second and third particles] from both rod ends. (b),(c),(e) The dark green and light green particles have attractive interactions. (d) The third (dark green) particles have attractive interactions with each other.

S1. EFFECTS OF HOOKS IN ACHIRAL RODS

To clarify the effects of the hook-shaped structure of the rods, we compared the results with the achiral protein rods with and without the hook particles [the rods shown in Fig. 1(a) and Fig. S1(a)]. Both of them exhibit the rod assembly and elliptical tube formation as shown in Fig. S2. The rod assemblies along the azimuthal (θ) and longitudinal (z) directions occur at larger rod curvatures $C_{\rm rod}$ for the rods with the hooks. This is caused by the excluded volume interaction of the hook particles; the rods without the hooks can form a more compact assembly on the elliptical tube [compare Fig. S2(b) with Fig. 2(f)].

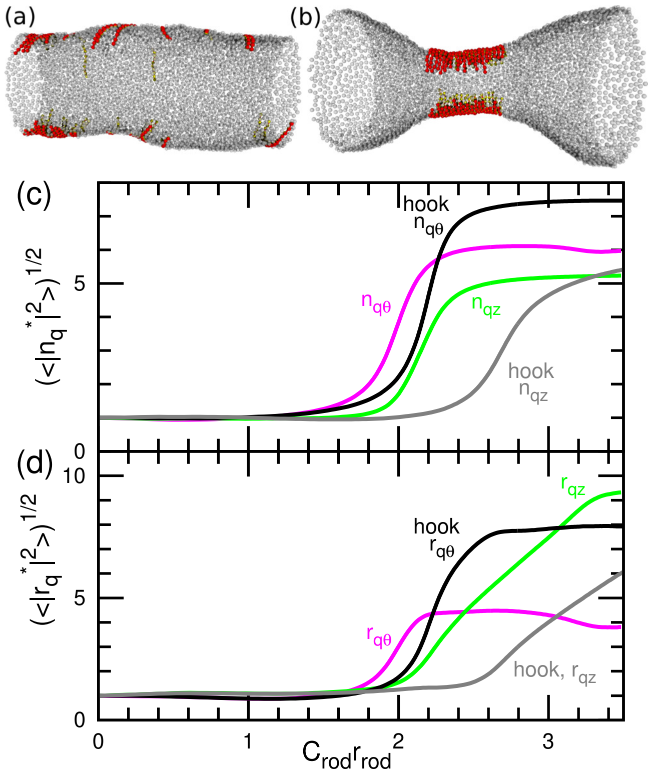

FIG. S2. Membrane tube deformation induced by achiral protein rods with and without the hooks at $R_{\rm cyl}/r_{\rm rod} = 1.31$ and $N = 4800$. No direct attraction is employed between the rods. (a),(b) Snapshots at (a) $C_{\rm rod}r_{\rm rod} = 2.1$, (b) 3.3 for the rod without the hooks. The membrane particles are displayed as transparent gray spheres. (c),(d) Fourier amplitudes of (c) rod densities and (d) membrane shapes calculated by REMD. The magenta and green lines represent the $q\theta$ and qz modes for the rods without the hooks, respectively. The black and gray lines represent the $q\theta$ and qz modes for the rods with the hooks, respectively.

S2. DEPENDENCE ON ATTRACTION SITES AND HOOK POSITION IN CHIRAL RODS

To confirm that our conclusions are robust to changes in the chiral protein structures, we examined the helical tube formation by four different types of the protein structures as shown in Figs. S1(b)–(e). The first three models have different segment pairs for the side-to-side attractive interaction, and in the last model, hook particles are connected with the second and third segments. All four models exhibit the helical cylinder formation as shown in Fig. S3. The pair of the third and fourth segments (and also the second and fourth segments) has a contact in the helical assembly of the original model so that the attraction strength ε for the assembly is close to that of the original model. In contrast, a greater value of ε is required for the attraction between the third segments, since they have no contact in the original rod assembly. The helical assembly can also be formed when the hook position is shifted to the inner position [hook

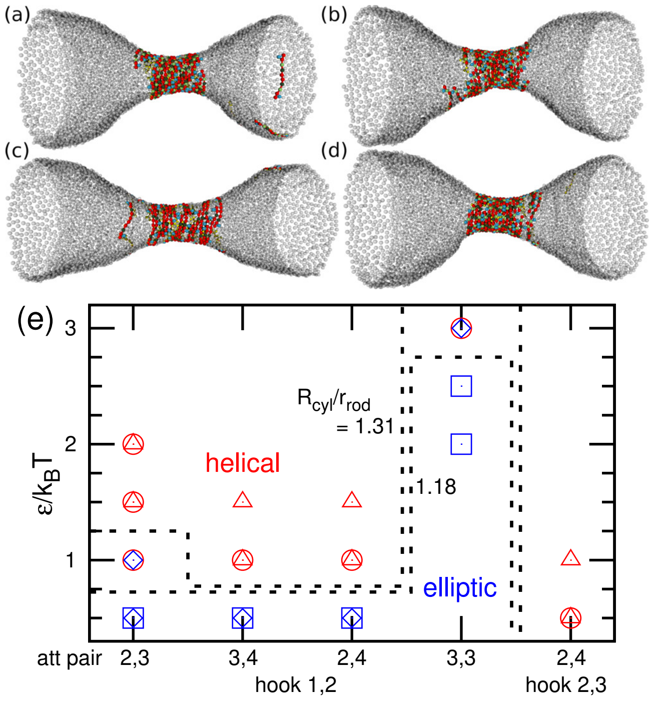

FIG. S3. helical tube formation of the chiral protein rods with the direct attraction between rods at $C_{\rm rod}r_{\rm rod} = 2.5$ and $N = 4800$. (a)–(d) Snapshots of membrane tubes at (a),(b),(d) $\varepsilon/k_{\rm B}T = 1$ and $R_{\rm cyl}/r_{\rm rod} = 1.31$ and at (c) $\varepsilon/k_{\rm B}T = 3$ and $R_{\rm cyl}/r_{\rm rod} = 1.18$. Snapshots in (a)–(d) are the results of the chiral rods depicted in Figs. S1(b)–(e), respectively. (e) Phase diagram for rod assembly of the elliptical tube and helical-cylinder shape. The open circles (triangles) and squares (diamonds) represent the elliptical and helical tubes at $R_{\rm cyl}/r_{\rm rod} = 1.18$ (1.31), respectively. The dashed lines are guides to the eye.

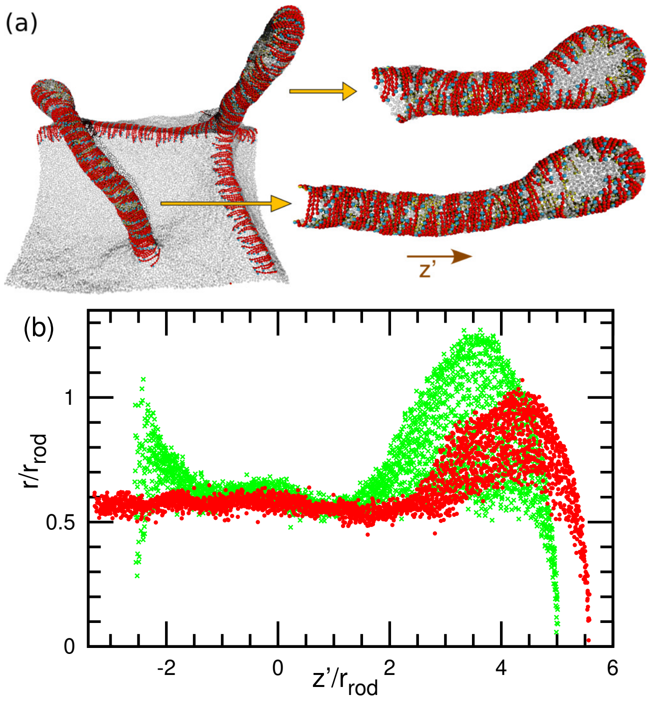

FIG. S4. Shape of the tubules protruded from a flat membrane at $\phi_{\rm rod} = 0.2$, $C_{\rm rod}r_{\rm rod} = 2.5$, and $t/\tau = 400$. (a) Snapshot of the entire membrane and extracted two tubules. (b) Radial distribution of the membrane particles of the tubules along the z' axis that is taken along the eigenvector of the greatest eigenvalue of the gyration tensor for the middle region of each tubule. The center of the cylindrical coordinate for the i-th particle is set to the center of the particles in the sliced region for $-0.2r_{\rm rod} < z' - z'_i < 0.2r_{\rm rod}$. The green crosses and rod dots represent the data corresponding to the upper and lower tubules shown in (a), respectively. The mean radius calculated from 12 tubules is shown in Fig. 3.

2,3, Fig. S3(d)]. Thus, the protein behavior does not qualitatively change under the above structure modifications. We conclude the helical assembly on a membrane is a general phenomenon for the chiral rods.

MOVIE CAPTIONS

Movie 1: Formation of the helical cylinder of chiral protein rods without direct attraction between the rods at $C_{\rm rod}r_{\rm rod} = 3.2$, $N = 4800$, and $R_{\rm cyl}/r_{\rm rod} = 1.18$. An equilibrium conformation at $C_{\rm rod}r_{\rm rod} = 2.8$ is used as an initial state.

Movie 2: Formation of the helical cylinder of chiral protein rods at $\varepsilon/k_{\rm B}T = 2$, $C_{\rm rod}r_{\rm rod} = 2$, $N = 9600$, and $R_{\rm cyl}/r_{\rm rod} = 1.31$. An equilibrium conformation at $\varepsilon/k_{\rm B}T = 0.5$ and $C_{\rm rod}r_{\rm rod} = 1.5$ is used as an initial state.

Movie 3: Tubulation from flat tensionless membrane induced by chiral protein rods at high rod density $\phi_{\rm rod} = 0.2$ and $C_{\rm rod}r_{\rm rod} = 2.5$. An equilibrium conformation at $C_{\rm rod}r_{\rm rod} = 0$ is used as an initial state.

Movie 4: Tubulation from flat tensionless membrane induced by chiral protein rods at low rod density $\phi_{\rm rod} = 0.05$ and $C_{\rm rod}r_{\rm rod} = 3$. An equilibrium conformation at $C_{\rm rod}r_{\rm rod} = 0$ is used as an initial state.
